# Comparing block characteristics of mixtures of short/intermediate- and long-acting local anesthetics for peripheral nerve block: a systematic review and meta-analysis

**DOI:** 10.1016/j.bjane.2025.844617

**Published:** 2025-03-28

**Authors:** Cheng Lin, Ana Larissa Guerrero, Joshua Jesin, Rohin Tangri, Nasong Anthony Luginaah, Kamal Kumar, Christopher Hansebout

**Affiliations:** a Western University, Schulich School of Medicine, London, Ontario, Canada; b University of Toronto, Department of Anesthesia, Toronto, Ontario, Canada

**Keywords:** Conduction anesthesia, Local anesthetic, Nerve block

## Abstract

**Introduction:**

Results from Randomized Controlled Trials (RCTs) on mixed Local Anesthetics (LA) are conflicting. We conducted a systematic review and meta-analysis on whether using mixed LA leads to faster onset of surgical block.

**Method:**

We conducted systemic review and meta-analysis of RCTs. Medline and Embase without language restriction from inception to June 15, 2024, were searched. Included RCTs had to compare mixed LA to long-acting LA in adult surgical patients for onset or duration of nerve blocks. Onset time to surgical block was the primary outcome. The Cochrane Risk of Bias Tool with GRADE methodology was utilized to assess evidence quality.

**Results:**

Nineteen trials including 1060 participants met the inclusion criteria. Mixed LA modestly reduced time to surgical block (-8.4 minutes; 95% CI -12.0 to -4.8 minutes; p = 0.0001; I^2^ = 0.99), sensory block duration (-226.2 minutes; 95% CI -352.2 to -100.1 minutes; p = 0.002; I^2^ = 0.98) and motor block duration (-259.2 minutes; 95% CI -399.5 to -119.0 minutes; p = 0.003; I^2^ = 0.98) but not time to analgesic request duration (-130.5 minutes; 95% CI -265.9 to 4.9 minutes; p = 0.057; I^2^ = 0.98). GRADE scoring ranged from low to very low.

**Conclusion:**

The existing evidence showed mixed LA led to a modest reduction in surgical block latency but also shortened block duration. Future studies should evaluate the role of mixed LA in lower limb blocks and optimal dosing of long-acting LA to balance onset latency and analgesic duration.

**PROSPERO registration:**

CRD42024552801.

## Introduction

Mixing short- or intermediate-acting local anesthetics with long-acting local anesthetics to achieve faster onset while attempting to preserve the duration of a Peripheral Nerve Block (PNB) dates to the 1950’s.^1^ Recent literature, including a meta-analysis suggests such practice yields modest or no improvement in block onset time, while significantly decreasing block duration.^2^, ^3^, ^4^ Several mechanisms for the reduction in block longevity have been proposed: vasodilatory effect of lidocaine facilitating the diffusion of long-acting local anesthetic away from the site of action or smaller total amount of long-acting local anesthetic administered in the mixture.^5^^,^^6^ This would seem to render the practice of mixing local anesthetic futile. Compounding this criticism, some authors cautioned against this practice, citing an elevated risk of Local Anesthetic Systemic Toxicity (LAST).^2^^,^^7^

Numerous Randomized Controlled Trials (RCTs) have attempted to clarify whether mixing short- and long-acting local anesthetics can lead to a faster onset. The elucidation of this outcome is important as there are ever-increasing demands for perioperative efficiency, while preserving the goal of providing prolonged postoperative analgesia. The recent meta-analysis focusing on ultrasound-guided blocks concluded that mixed local anesthetics had no effect on block latency while shortening block duration.^4^ These reviews excluded stimulator-guided blocks and one RCT published after the study protocol.^8^ Further, the authors did not explore the effects of differences in local anesthetic regimens (e.g., lidocaine vs. mepivacaine, doses, use of epinephrine etc.) on the block outcomes. While this meta-analysis provided important insight into mixing local anesthetics, there remained some salient detail to be elucidated, such as dosing, choice of local anesthetics and location of blocks. We designed this systematic review and meta-analysis to consolidate the evidence on the effects of mixing short- or intermediate-acting local anesthetics with long-acting local anesthetics on single shot peripheral nerve blocks. We hypothesized that using a mixture of short- or intermediate- and long-acting local anesthetics will lead to faster block onset.

## Methods

This systematic review and meta-analysis complies with the PRISMA statement and is registered on PROSPERO (CRD42024552801). The clinical question, study inclusion criteria, outcomes and analysis plans were defined *a priori*.

### Literature search

We searched Ovid MEDLINE and Embase from inception to June 15, 2024, for RCTs, without language restriction. The full search strategy is available in the Appendix (Supplemental Digital Content 1). The reference lists of included articles were manually searched for additional studies. Included studies met the following criteria:

#### Study selection criteria

##### Population

Studies had to recruit adult patients who received PNBs and who underwent surgery. Studies investigating subjects without surgery (e.g., healthy volunteers) were excluded. Neuraxial blocks and local infiltration anesthesia were further excluded.

##### Intervention and control

Included RCTs compared single-shot blocks using a mixture of short- or intermediate-acting local anesthetics (e.g., prilocaine, chloroprocaine, lidocaine or mepivacaine) and long-acting local anesthetics (e.g., tetracaine, etidocaine, ropivacaine, levobupivacaine or bupivacaine) to long-acting local anesthetics alone. These two arms would hereafter be referred to as the mixture group and the long-acting only group in this review. Adjuvants could be included if there still was a comparison between a mixture and long-acting anesthetic. Study arms including only short- or intermediate- acting local anesthetics, liposomal bupivacaine, or a mixture of two long-acting local anesthetics were excluded from the analysis.

##### Outcomes

Specific outcome definitions are listed in Table 1. The primary outcome is the surgical block latency, which pooled the following outcomes in this priority sequence: composite block, complete sensory block latency followed by complete motor block latency, if the prior time was not reported. Secondary outcomes included early sensory block latency, early motor block latency, composite block latency, complete sensory block latency, complete motor block latency, the duration of sensory block, motor block and analgesic duration. Analgesic outcomes such as pain scores or analgesic consumption were recorded. Safety outcomes included any plasma local anesthetic level, LAST and neurologic deficit. Quality outcomes included block failure (needing supplement or conversion to general anesthesia), intraoperative sedation requirement and satisfaction.

**Table 1:** Outcomes and their definitions.

Outcomes	Definitions
Surgical block	Surgery ready block, including a composite of motor and sensory deficit, complete sensory or motor block
Surgical block latency	Time to achieve surgical block
Composite block	A composite of both motor and sensory block
Composite block latency	Time to achieve composite block
Early sensory block	Earliest presence of any sensory deficit
Early sensory block latency	Time to achieve early sensory block
Complete sensory block	A sensory block beyond early sensory block. Ex. dullness or insensate to touch, pinprick or cold
Complete sensory block latency	Time to achieve complete sensory block
Early Motor block	Earliest presence of any motor deficit
Motor block onset latency	Time to achieve early motor block
Complete motor block	A motor block beyond early motor block. Ex. Profound weakness to absent motion
Complete motor block latency	Time to achieve complete motor block
Analgesic duration	Time to onset of pain, analgesic request or a prespecified pain score, as defined by each study

##### Article selection and data extraction

All titles, abstracts, and full texts (if required to assess the article for inclusion) were reviewed in duplicate and any disagreement resolved by consensus. Covidence.org was utilized for screening and data extraction using the same process for disagreement. To aid the meta-analysis, we followed the methods and suggestions of the Cochrane Handbook for Systematic Reviews of Interventions.^9^ Medians, interquartile ranges and range values were approximated into means and their corresponding standard deviation. Pooled averages and standard deviations were calculated for studies with multiple arms (e.g., different doses of short acting-local anesthetics, different short acting-local anesthetics) to combine them into a single group. For studies where patients received multiple blocks and outcomes were measured separately (e.g., femoral, and sciatic blocks), the number of subjects was divided by the number of measurements (e.g., n/2 for femoral and sciatic) while keeping the original mean and standard deviation.

##### Risk of bias evaluation

The Cochrane Risk of Bias 2 (RoB 2) tool was used to assess each study's risk of bias, based on the 5 domains: (1) Randomization process, (2) Deviation from intended interventions, (3) Missing outcome data, (4) Measurement of the outcome and (5) Selection of the reported result.

##### Level of evidence evaluation

The Grading of Recommendations Assessment, Development and Evaluation (GRADE) guideline was used to evaluate the certainty of evidence and level of recommendation for each outcome in the meta-analysis.

### Statistical analysis

Statistical analysis was done using *R* (version 4.4.1, the R Foundation for Statistical Computing, Vienna). Standard summary measures were generated with the Mean Difference (MD) for continuous data and Odds Ratios (ORs) for categorical data with their corresponding 95% Confidence Intervals (95% CIs) and an α = 0.05. All analyses were done using a random-effects model. I^2^ statistic was used to quantify heterogeneity. An I^2^ value of 0% to 25% was considered low, 25% to 50% as moderate, and greater than 50% as high heterogeneity. The Mantel-Haenszel method without continuity correction was used for zero event studies. To determine whether the differences in local anesthetic used can predict block latency and duration, we utilized multiple meta-regression using the maximum likelihood estimation, with the following as co-variates: log-transformed ratio of total potency of the mixture (P_MT_) to long-acting only (P_LA_), potency of the short-acting local of the mixture (P_MS_) to P_LA_ and potency of the long-acting local of the mixture (P_ML_) to P_LA_.^10^ Collinearity of the ratios was examined using intercorrelation matrix and highly correlated ratios were excluded. Best model was determined using Anova.

A funnel plot was constructed for the primary outcome and Egger regression was used to investigate for statistical evidence of publication bias.

All subgroup analyses were planned *a priori*. These included block technique (ultrasound vs. nerve stimulator vs. landmark), use of adjuvants (e.g., epinephrine), block location (upper vs. lower limb), local anesthetic used (e.g., ropivacaine vs. bupivacaine, lidocaine vs. mepivacaine), by country (developed vs. developing) and risk of bias (low, some concerns and high risk). Subgroup analysis on duration of sensory, motor block and analgesia were also planned for dose of long-acting local anesthetics in the mixture (i.e., same dose as in the long-acting local only group vs. reduced dose) and use of adjuvants.

For outcomes with high heterogeneity, outlier and influential analyses were conducted and results summarized by excluding such studies. Sensitivity analysis on secondary outcomes with high heterogeneity was examined using subgroups to determine the source of heterogeneity.

## Results

### Literature search and study selection

**Figure 1:**
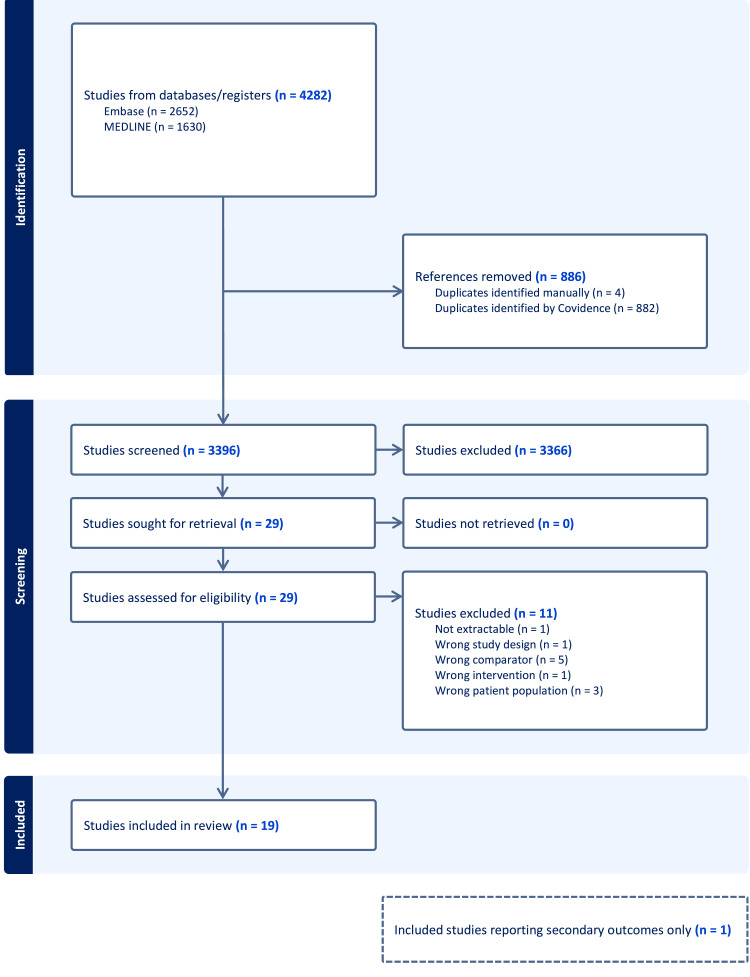
PRISMA flowsheet describing the study selection process. MEDLINE and EMBASE were searched for relevant articles and screened against our inclusion criteria.

Our search strategy yielded 4282 studies with 19 RCTs, with 1060 participants, ultimately meeting the inclusion criteria (Fig. 1).^2^^,^^3^^,^^5^^,^^6^^,^^8^^,^^11^, ^12^, ^13^, ^14^, ^15^, ^16^, ^17^, ^18^, ^19^, ^20^, ^21^, ^22^, ^23^, ^24^ One included study with 34 patients reported duration of motor block and analgesia, but not the primary outcome.^13^ Eleven studies were excluded during the full text review: 5 studies did not include a long-acting local anesthetic comparator group,^25^, ^26^, ^27^, ^28^, ^29^ 3 studies did not include a surgical population,^30^, ^31^, ^32^ one study used liposomal bupivacaine,^33^ one study was not an RCT,^34^ and one study did not present data in an extractable format.^35^

### Study characteristics

**Figure 2:**
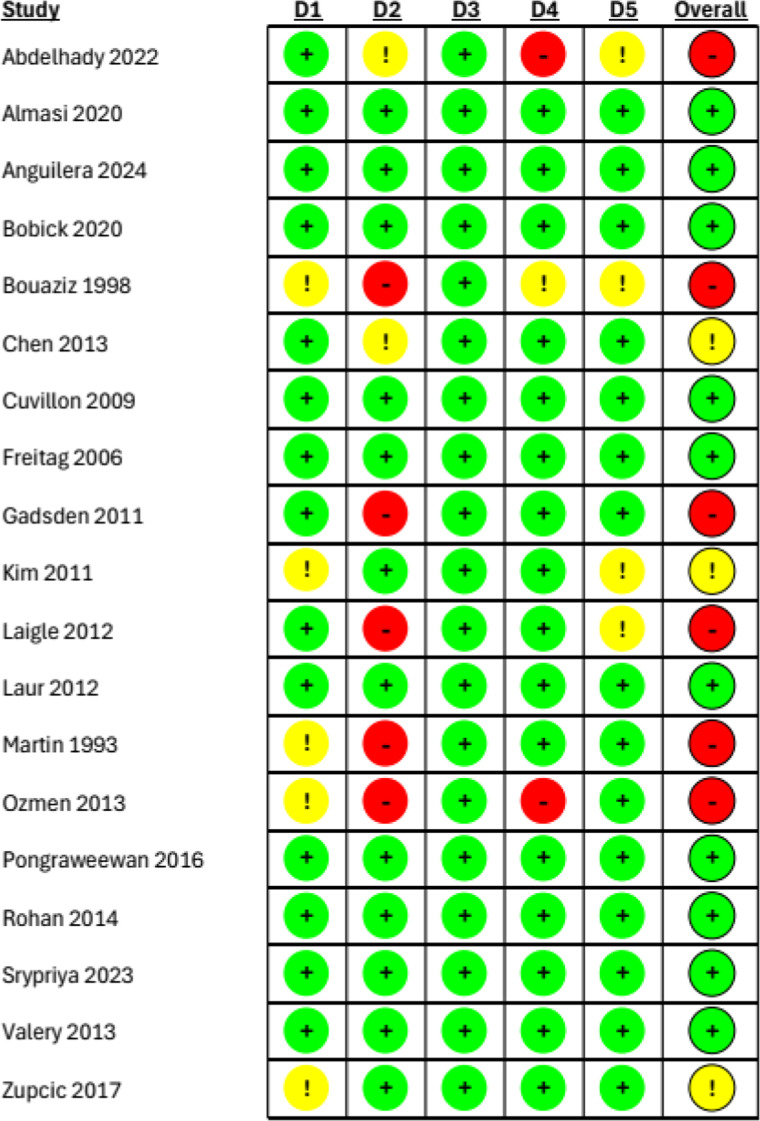
Risk of Bias Assessment. For each domain of the Cochrane Risk of Bias Tool, green inidcate low risk of bias, yellow indicate some concerns and red indicates high risk of bias. D1, Randomization process; D2, Deviations from intended interventions; D3, Missing outcome data; D4, Measurement of the outcome; D5, Selection of the reported result.

Overall, the studies were heterogeneous (Table 2). Reported outcomes by study are shown in a Supplementary Table (Supplemental Digital Content 2). Fourteen studies targeted the brachial plexus,^2^^,^^3^^,^^8^^,^^11^, ^12^, ^13^^,^^15^^,^^16^^,^^18^, ^19^, ^20^, ^21^, ^22^, ^23^ 4 the lower limb,^5^^,^^6^^,^^14^^,^^17^ and one assessed paravertebral block.^24^ Eight studies used nerve stimulator only,^5^^,^^13^, ^14^, ^15^^,^^18^, ^19^, ^20^^,^^22^ 5 used ultrasound only,^3^^,^^8^^,^^11^^,^^16^^,^^17^^,^^23^ and 6 used both modalities.^2^^,^^6^^,^^12^^,^^21^^,^^24^ All studies used amide local anesthetics except for 1 study where chloroprocaine was used.^16^ Eight studies used epinephrine,^8^^,^^12^^,^^14^^,^^18^, ^19^, ^20^^,^^22^^,^^23^ of which 2 studies added epinephrine to the mixture group only.^18^^,^^22^ Most studies, when compounding the mixture, decreased the dose of long-acting local anesthetic by half when compared to the long-acting only group. Four studies used the same amount of long-acting local anesthetic in the long-acting local only and the mixture groups.^5^^,^^6^^,^^11^^,^^16^ One trial had a group with long-acting local anesthetic with clonidine adjuvant; this arm was excluded from analysis.^22^ Ten studies were graded low risk of bias,^6^^,^^8^^,^^11^^,^^12^^,^^14^^,^^15^^,^^18^^,^^21^, ^22^, ^23^ 3 with some concerns,^5^^,^^13^^,^^16^^,^^24^ and the rest were ranked high risk (Fig. 2).^2^^,^^3^^,^^13^^,^^17^^,^^19^^,^^20^ Two studies were conference abstracts.^16^^,^^17^

**Table 2:** Study characteristics of included trials.

Demographic					Block Detail		Local Anesthetics			
Trial	Year	Total (n)	Outcome analyzed	Surgery	Type of Block	Technique	Long-Acting Local Anesthetics^a^	Mixture of Local Anesthetics^a^	Adjuvants^b^	Risk of Bias
Abdelhady et al.	2022	66	Insensate to pinprick	Brachiocephalic fistula	Axillary	U/S	Bupivacaine 0.5% 30 mL	Bupivacaine 0.5% 15 mL		High
								Lidocaine 2% 15 mL		
Almasi et al.	2020	87	Insensate to touch	Hand and forearm	Axillary	U/S	Bupivacaine 0.5% 20 mL	1. Bupivacaine 0.5% 15 mL		Low
								Lidocaine 2% 15 mL		
							Saline 10 mL	2. Bupivacaine 0.5% 20 mL		
								Lidocaine 2% 10 mL		
Aguilera et al.	2024	40	≥ 14/16 scale of motor-sensory block	Forearm, wrist and hand	Infraclavicular	U/S	Bupivacaine 0.5% 35 mL^c^	Bupivacaine 0.5% 17.5 mL	Epinephrine 1:200,000	Low
								Lidocaine 2% 17.5 mL^c^		
Bobik et al.	2020	63	Dullness to pinprick	Forearm and hand	Axillary	U/S + Stim	1. Bupivacaine 0.375% 30 mL^c^	Bupivacaine 0.5% 15 mL	Epinephrine 1:200,000	Low
							2. Ropivacaine 0.5% 30 mL	Lidocaine 2% 15 mL^c^		
Bouaziz et al.	1998	34	Duration of block^g^	Hand	Midhumeral	Stim	Bupivacaine 0.5% 20 mL	Bupivacaine 0.5% 10 mL		High
								Lidocaine 2% 10 mL		
Chen et al.	2013	60	Insensate to pinprick	Knee	Sciatic	Stim	Ropivacaine 0.75% 10 mL	Ropivacaine 0.75% 10 mL		Some concerns
							Saline 10 mL	Lidocaine 2% 10 mL		
Cuvillon et al.	2009	82	Surgery ready^f^	Lower limb	Femoral and Sciatic	Stim	1. Bupivacaine 0.5% 40 mL^c^^,^^d^	1. Bupivacaine 0.5% 20 mL	Epinephrine 1:200,000	Low
								Lidocaine 2% 20 mL^c^^,^^d^		
							2. Ropivacaine 0.5% 40 mL^c^^,^^d^	2. Ropivacaine 0.5% 20 mL		
								Lidocaine 2% 20 mL^c^^,^^d^		
Freitag et al.	2006	96	Insensate to pinprick	Forearm, wrist and hand	Axillary	Stim	Ropivacaine 0.75% 40 mL	1. Ropivacaine 0.75% 10 mL		Low
								Prilocaine 1% 30 mL		
								2. Ropivacaine 0.75% 20 mL		
								Prilocaine 1% 20 mL		
Gadsden et al.	2011	69	Insensate to pinprick	Shoulder arthroscopy	Interscalene	U/S + Stim	Bupivacaine 0.5% 30 mL	Bupivacaine 0.5% 15 mL		High
								Mepivacaine 1.5% 15 mL		
Kim et al.	2011	60	Complete loss of sensation	Upper extremity	Supraclavicular	U/S	Ropivacaine 1% 20 mL	Ropivacaine 1% 20 mL	Epinephrine 1:200,000	Some concerns
							Saline 10 mL^c^	Chloroprocaine 3% 10 mL^c^		
Laigle et al.	2012	30	Surgery ready^f^	Foot	Sciatic	U/S	Ropivacaine 0.75% 30 mL	Ropivacaine 0.75% 15 mL		High
								Mepivacaine 1.5% 15 mL		
Laur et al.	2012	95	Reduced sensation to cold	Distal upper extremity	Infraclavicular	Stim	Bupivacaine 0.5% 40 mL	Bupivacaine 0.5% 20 mL	Epinephrine 1:200,000	Low
								Mepivacaine 1.5% 20 mL^c^		
Martin et al.	1993	60	Dullness to pinprick	Upper limb distal to shoulder	Axillary	Stim	Bupivacaine 0.25% 44 mL^c^	Bupivacaine 0.25% 22 mL	Epinephrine 1:400,000	Some concerns
								Lidocaine 1% 22 mL^c^		
Ozmen et al.	2013	120	Surgery ready^f^	Forearm or hand	Infraclavicular	Stim	Bupivacaine 0.5% 20 mL	Bupivacaine 0.5% 10 mL		Some concerns
								Lidocaine 2% 10 mL		
Pongraweewan et al.	2016	90	Insensate to pinprick	Arteriovenous fistula	Infraclavicular	U/S + Stim	Bupivacaine 0.5% 30 mL	Bupivacaine 0.5% 20 mL		Low
								Lidocaine 2% 10 mL		
Rohan et al.	2014	75	Complete loss of sensation	Upper extremity	Supraclavicular	Stim	Ropivacaine 0.75% 20 mL	Ropivacaine 0.75% 20 mL	Epinephrine 1:200,000	Low
							Saline 10 mL	Lidocaine 2% 10 mL^c^		
Sripriya et al.	2023	63	16/16 scale sensory motor block	Upper extremity	Supraclavicular	U/S	Bupivacaine 0.5% 20 mL^c^	Bupivacaine 0.5% 10 mL	Epinephrine 1:200,000	Low
								Lidocaine 2% 10 mL^c^		
Valery et al.	2013	60	Complete loss of sensation	Leg	Sciatic	U/S + Sim	1. Ropivacaine 0.75% 5 mL	Ropivacaine 0.75% 5 mL		Low
							2. Ropivacaine 0.75% 10 mL	Lidocaine 1% 5 mL		
Zupcic et al.	2017	85	Change to pinprick and temperature	Breast and axillary node	Paravertebral T2-T4	U/S + Stim	Levobupivacine 0.5% 21 mL^e^	Levobupivacine 0.5% 14 mL		Some concerns
								Lidocaine 2% 7 mL^e^		

a Pre-mixed local anesthetic concentration and volume.

b Final concentration of adjuvant in the mixture.

c Adjuvant added.

d Twenty mL at femoral and 20 mL at sciatic nerve.

e Seven mL per paravertebral level

f Endpoint of measurement not clearly defined.

g Study not included for primary outcome analysis. U/S, Ultrasound; U/S + Stim, Ultrasound and Stimulator; Stim, Stimulator.

### Primary outcome: surgical block latency

**Figure 3:**
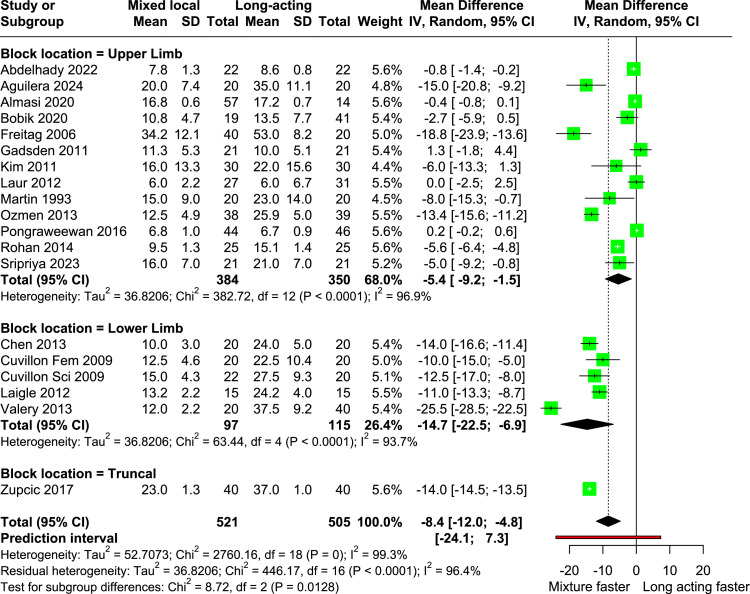
Forest plot of onset time to surgical block. Data are grouped by block locations.

The primary outcome and its subgroup analyses are listed in a Supplementary Table (Supplemental Digital Content 3). Patients receiving a mix of short- or intermediate- and long-acting local anesthetics experienced 8.4 minutes (95% CI -12.0 to -4.8 minutes; p = 0.0001; I^2^ = 0.99) reduced surgical block latency (Fig. 3). Likewise, decreased surgical block latencies were observed in all preplanned subgroups, except when mepivacaine was used in the mixture group (-4.4 minutes [95% CI -20.7 to 11.9 minutes]; p = 0.37; I^2^ = 0.961) and in studies with high risk of bias (-6.3 minutes [95% CI -14.4 to 1.7 minutes]; p = 0.095; I^2^ = 0.978). Meta-regression analysis did not identify any potency ratios as a predictor of surgical block latency. In upper limb block, the variation in potency was found to contribute 28.2% of heterogeneity. Publication year was also not a significant predictor for surgical block latency. Eight studies were identified as outliers^2^^,^^3^^,^^6^^,^^11^^,^^15^^,^^18^^,^^21^^,^^24^^]^ and 5 as influential,^6^^,^^11^^,^^19^^,^^21^^,^^24^ and when excluded, neither affected the outcomes or lowered heterogeneity (without outliers: -9.4 minutes [95% CI -12.3 to -6.6 minutes]; p < 0.0001, I^2^ = 0.907; without influential studies: -7.9 minutes [95% CI -11.6 to -4.3 minutes]; p < 0.001, I^2^ = 0.964).

### Secondary outcomes

Secondary outcomes and their subgroup analyses are detailed in a Supplementary Table (Supplemental Digital Content 4).

### Sensory and motor block latency

Five studies measured composite block latency. It was reduced by 11.1 minutes (95% CI -14.6 to -7.7 min; p = 0.004; I^2^ = 0.642) in the mixture group. Complete sensory block latency was reported by 14 studies and the pooled result showed a reduction of 7.8 min (95% CI -12.6 to -3.0 min; p = 0.004; I^2^ = 0.99).^2^^,^^3^^,^^5^^,^^6^^,^^11^^,^^12^^,^^15^, ^16^, ^17^, ^18^, ^19^, ^20^, ^21^, ^22^^,^^24^ Six studies measured early sensory block latency.^3^^,^^19^, ^20^, ^21^, ^22^^,^^24^ This time was decreased by 3.7 minutes (95% CI -6.7 to -0.6 min; p = 0.027; I^2^ = 0.99) in the mixture group. Seven studies reported complete motor block latency (-10.4 min; 95% CI -23.4 to 2.6 min; p = 0.098; I^2^ = 0.99),^2^^,^^6^^,^^11^^,^^15^^,^^16^^,^^18^^,^^22^ and 3 studies measured early motor block latency (-1.3 min; 95% CI -8.4 to 5.7 min; p = 0.50; I^2^ = 0.98).^3^^,^^21^^,^^22^ Both were not statistically different when compared to long-acting only group. Sensitivity analyses were carried out given the high heterogeneity, but no subgroup significantly contributed to heterogeneity. Composite block latency, early sensory and early motor block latency were reduced without ultrasound use. Meta-regression with potency ratios tested did not identify heterogeneity contributor or predictor to sensory and motor block latency.

### Duration of sensory, motor block and analgesia

Twelve trials studied the duration of sensory block.^3^^,^^5^^,^^8^^,^^11^^,^^12^^,^^14^^,^^15^^,^^17^, ^18^, ^19^^,^^21^^,^^22^ Mixed local anesthetics decreased the duration by 226.2 minutes (95% CI -352.20 to -100.13 min; p = 0.002; I^2^ = 0.98). Ten studies examined the duration of motor blockade; it was reduced by 259 minutes (95% CI -399.49 to -118.95 min; p = 0.0021; I^2^ = 0.98).^2^^,^^3^^,^^8^^,^^12^, ^13^, ^14^, ^15^^,^^18^^,^^21^^,^^22^ The analgesic duration was reported by 11 studies: 4 defined as request of analgesia,^8^^,^^14^^,^^20^^,^^22^, 6 as onset of pain^2^^,^^3^^,^^6^^,^^13^^,^^16^^,^^23^^]^ and 1 as time to moderate pain.^24^ The change in analgesic duration in the mixture group did not reach statistical significance (-130.5 min; 95% CI -265.93 to 4.94 min; p = 0.057; I^2^ = 0.98). As for the subgroup analysis, the duration of sensory blockade was not significantly different in the mixture group when compared to the long-acting only group when the long-acting local anesthetic dose was the same in both groups (-80.3 min; 95% CI -299.65 to 139.05 min; p = 0.25; I^2^ = 0.98) but reduced when the dose was lower (-271.7 min; 95% CI -427.96 to -115.53 min; p < 0.0001; I^2^ = 0.97).^5^^,^^11^^,^^22^ Likewise, three studies examining the duration of analgesia were included in this subgroup analysis.^6^^,^^16^^,^^22^ There was no statistically significant change in the analgesic duration when the long-acting local anesthetic dose in the mixture group was the same (-51.4 min; 95% CI -281.90 to 179.11 min; p = 0.44; I^2^ = 0.83) or lower (-165.1 min; 95% CI -358.54 to 28.38 min; p = 0.083; I^2^ = 0.96) in the long-acting only group.^6^^,^^16^^,^^22^ Meta-regression similarly demonstrated potency ratio P_ML_/P_LA_ as a predictor for sensory (p = 0.010) and analgesic duration (p = 0.027), with higher potency correlated with longer duration. This variation in long-acting local anesthetic in the mixture accounted for 42.0% and 55.1% of heterogeneity in sensory and motor block duration. Lidocaine but not mepivacaine was shown to reduce sensory and analgesic duration. As for long-acting local anesthetic, bupivacaine but not ropivacaine had reduced analgesic duration. Subgroup analysis by epinephrine use showed adding epinephrine led to a reduction in sensory block duration (epinephrine, -304.3 min; 95% CI -498.60 to -110.10 min; p = 0.009; I^2^ = 0.96); there was no statistical difference in duration in epinephrine-free studies (-142.5 min; 95% CI -338.09 to 52.99 min; p = 0.12; I^2^ = 0.98). A similar trend was also observed in the motor block duration with epinephrine (-309.9 min; 95% CI -540.99 to -78.89 min; p = 0.018; I^2^ = 0.97) and without (-198.9 min; 95% CI -447.32 to 49.59 min; p = 0.09; I^2^ = 0.98). Neither subgroup reached statistical significance in analgesic duration (epinephrine, -214.6 min; 95% CI -702.95 to 273.68 min; p = 0.12; I^2^ = 0.97); and no epinephrine, (-87.0 min; 95% CI -204.92 to 30.95 min; p = 0.26; I^2^ = 0.94). Subgroup analysis by the definition of analgesic duration showed a statistically significant reduction only with onset of pain (-131.1 minutes; 95% CI -241.1 to -21.3 minutes; p = 0.028; I^2^ = 0.948). Sensitivity analysis failed to identify any subgroup with significant contribution to heterogeneity.

### Block success and quality

Block success was reported by 11 studies.^5^^,^^8^^,^^11^^,^^12^^,^^14^^,^^15^^,^^17^^,^^18^^,^^20^^,^^21^^,^^23^ There was no difference between the failure rate of both groups (OR = 0.68; 95% CI 0.22–2.16; p = 0.46; I^2^ = 0.39). Four studies measured the amount of intraoperative sedation requirement, but the reported format for this outcome was too heterogenous for meta-analysis.^5^^,^^11^^,^^20^^,^^24^ However, no difference was detected by the individual studies. One study did not find any difference between surgeon and patient satisfaction between the two local anesthetic solutions used.^21^

### Other pain-related outcomes

Two studies recorded the amount of postoperative morphine use and individually did not demonstrate any difference between the two arms.^14^^,^^17^ One study reported more patients needed diclofenac in the first 12 hours in the mixture group,^24^ but another study demonstrated no difference in diclofenac use.^11^ Pain scores were reported by 7 studies but there were not enough congruent time points to perform meta-analysis.^2^^,^^8^^,^^14^^,^^15^^,^^19^^,^^21^^,^^24^ Two studies reported statistically significant, albeit clinically insignificant differences favoring long-acting only group. One documented a difference at 3 hours (1 ± 0.74 vs. 2 ± 1.48 on an 11-point numeric rating scale),^24^ and another study at 8 hours (1 ± 0.74 and 2 ± 1.49).^3^ Neither detected any difference before or after the above time points, respectively.

### Block complications

No cardiac or severe neurologic LAST was reported in any study. Two studies performed serial plasma concentration measurements of local anesthetics. One used the same dose of ropivacaine in both groups and found the maximum plasma Concentration (C_max_) was significantly higher in the mixture group (3110 ± 1100 ng.mL^-1^ vs. 2600 ± 900 ng.mL^-1^).^5^ In the other study, the dose of long-acting local anesthetic was halved in the mixture and the C_max_ for ropivacaine was lower from 1840 ± 590 ng.mL^-1^ to 460 ± 270 ng.mL^-1^ and bupivacaine from 1095 ± 520 ng.mL^-1^ to 450 ± 80 ng.mL^-1^ when the mixture was used.^14^ Four studies tracked permanent neurologic deficit during the study period, none were reported.^8^^,^^20^^,^^21^^,^^23^ Hemodynamic parameters were measured in five studies, but the reported format did not permit meta-analysis. Three studies did not detect any difference in heart rate, oxygen saturation or blood pressure,^5^^,^^8^^,^^15^ one study recorded higher stroke volume variation, more frequent hypotension and higher fluid requirement in the mixture group,^24^ and one study recorded one episode of significant bradycardia in the long-acting local anesthetic group.^3^

### Publication bias

While Egger regression did not demonstrate asymmetry (p = 0.23), the contoured funnel plot (Supplemental Digital Content 5) demonstrated a high number of studies with standard error greater than 2 and a majority of studies with high statistical significance (p < 0.01). These suggested results may be skewed by small-study effects, and publication bias could not be excluded.

### Certainty of evidence and level of recommendation

The summary assessment is presented in Table 3. Due to high heterogeneity and small number of studies available for some secondary outcomes, the evidence certainty ranked from low to very low.

**Table 3:** Summary of findings table.

Mixed local anesthetic compared to long-acting local anesthetic only for peripheral nerve block				
	Anticipated absolute effects (95% CI)		N° of participants (studies)	Certainty of the evidence (GRADE)
Outcomes	Long-acting local anesthetic only	Mixed local anesthetic		
Surgical block latency	The mean time to surgical block ranged from 6–38 min	MD 8.4 min lower (12 lower to 4.8 lower)	1026 (18 RCTs)	⨁⨁◯◯ Low^a^
Complete block latency	The mean time to surgical block ranged from 6–38 min	MD 11.1 min lower (14.6 lower to 7.7 lower)	271 (5 RCTs)	⨁⨁◯◯ Low^a^
Complete sensory block latency	The mean time to complete sensory block was 6–38 min	MD 7.8 min lower (12.6 lower to 3.0 lower)	561 (10 RCTs)	⨁⨁◯◯ Low^a^
Complete motor block latency	The mean time to complete motor block was 7–65 min	MD 10.4 min lower (23.4 lower to 2.6 higher)	401 (7 RCTs)	⨁⨁◯◯ Low^a^
Early sensory block latency	The mean onset of any sensory block was 2–23 min	MD 3.7 min lower (6.7 lower to 0.6 lower)	381 (6 RCTs)	⨁◯◯◯ Very low^a^^,^^b^^,^
Early motor block latency	The mean onset of any motor block was 6–10 min	MD 1.3 min lower (8.4 lower to 5.7 higher)	184 (3 RCTs)	⨁◯◯◯ Very low^a^^,^^b^^,^^c^
Duration of sensory block	The mean duration of sensory block was 227–1758 min	MD 226.2 min lower (352.2 lower to 100.1 lower)	657 (12 RCTs)	⨁⨁◯◯ Low^a^
Duration of motor block	The mean duration of motor block was 466–1704 min	MD 259.2 min lower (399.5 lower to 119 lower)	551 (10 RCTs)	⨁⨁◯◯ Low^a^
Duration of analgesia	The mean duration of analgesia was 264–2298 min	MD 130.5 min lower (265.3 lower to 4.9 higher)	591 (11 RCTs)	⨁⨁◯◯ Low^a^

CI, Confidence Interval; MD, Mean Difference.

a Very high heterogeneity.

b Majority of studies ranked as high risk of bias.

c Low number of studies.

## Discussion

Using mixed local anesthetics in peripheral nerve blockade remains a controversial yet common practice. Our meta-analysis demonstrated a modest reduction in surgical block latency of 8.4 minutes (95% CI -12.0 to -4.8 min) at the expense of reduced sensory block duration (-226.2 minutes, 95% CI -352.20 to -100.13 min), motor block duration (-259 minutes, 95% CI -399.49 to -118.95 min) but not analgesic duration (-130.5 minutes; 95% CI -265.93 to 4.94 min). Pietroski dos Santos et al. in their meta-analysis concluded that mixed local anesthetics did not reduce sensory block latency as a primary outcome.^4^ This agreed with our finding where the use of mixed local anesthetic did not shorten complete sensory block latency in the ultrasound subgroup (Supplementary Content 3). Like our findings, their study identified a reduced sensory block duration but no difference in pain score, opioid consumption, block failure or complications. A notable difference is that we found a reduction in motor block duration in the ultrasound subgroup whereas Pietroski dos Santos et al. did not. However, the upper-bound of the 95% CI was just -1.8 min. The difference may be due to the inclusion of an additional study in our analysis.^9^

Safety is a primary concern when mixing local anesthetics as there is higher risk of drug error and potential for LAST. No studies reported significant LAST or persistent neurologic injury. However, one study reported elevated plasma levels of ropivacaine when co-administered with lidocaine, attributing this to the vasodilatory effect of lidocaine; no clinical symptoms were recorded.^5^ The safety of mixing local anesthetics remains debated, with a case report of significant LAST when mixed local anesthetics was used.^2^^,^^7^^,^^36^ Therefore, one must exercise caution, assuming at least additive toxic effect of individual local anesthetics when using a mixture.

A wide variety of types and doses of local anesthetics were used in the studies. Lidocaine but not mepivacaine reduced surgical block and complete sensory block latency. However, mepivacaine was only examined by 3 studies. Most studies used 2% lidocaine and 1.5% mepivacaine which may not be equipotent. Neither local anesthetic potencies nor the specific long-acting local anesthetic appeared to predict block latency. Sensory and analgesic duration were reduced when lidocaine or ropivacaine was used in the mixture. Such difference may be explained by the more prominent vasodilation of lidocaine over mepivacaine and increased protein binding of bupivacaine over ropivacaine.^37^ Further, there is a positive correlation between the long-acting local anesthetic potency in the mixture and sensory and analgesic duration.

Several studies attempted to overcome the decreased block duration by utilizing epinephrine.^8^^,^^23^ Interestingly, in our analysis, epinephrine appeared to mediate a reduction in block duration. However, careful examination of the data demonstrates that, while epinephrine prolonged the effect of both the mixture and long-acting anesthetics, this effect was more pronounced on the long-acting agents alone. The block duration of epinephrine-containing and epinphrine-free mixture was comparable. Further, there was no in-between group difference identified on all subgroup analyses concerning epinephrine use.

Our review identified areas that warrant further investigation. While the reduction brachial plexus block latency was modest with mixed local anesthetics, lower limb blocks showed a more meaningful reduction in latency of 14.7 minutes (P_subgroup_ = 0.013). It is possible the larger surface area of the brachial plexus permits more local anesthetic exposure compared to the terminal nerve of femoral and sciatic, thereby, reducing the effect of the mixture. As there are only 4 studies investigating lower limb, more studies would consolidate the understanding on the effect of using mixed local anesthetics for lower limbs blocks. Another finding is the positive correlation between the relative potency of long-acting local anesthetic in the mixture and long-acting only groups with sensory and analgesic duration. Studies using varying doses of short- and long-acting local anesthetics versus latency and duration would also provide insight into the optimal regimen in different clinical scenarios.

### Implications for practice

In their meta-analysis, Pietroski dos Santos et al. concluded mixed local anesthetics should not be used to reduce block latency.^4^ However, inclusion of additional studies in our review identified circumstances where using mixed local anesthetics may be advantageous. In an austere environment where an ultrasound is unavailable, using mixed local anesthetics can improve block latency. Likewise, despite ultrasound-guidance, the needling of a novice or infrequent block performer may mimic the imprecision of stimulator-based technique, and these practitioners may reap more benefit from using mixed local anesthetics. Mixed local anesthetics also showed more pronounced reduction in block latency in lower limb blocks. In contrast, with experienced providers, in surgery where significant pain is expected and prolonged block duration desirable, mixed local anesthetics should be avoided. Reassuringly, despite the shortened block duration, pooled differences in analgesic duration did not reach statistical significance and individual studies did not report a meaningful difference in pain related outcomes.^38^

Our subgroup analyses and meta-regression provided some insight to optimizing mixed local anesthetic regimen. Lidocaine but not mepivacaine should be used to reduce block latency. Ropivacaine may be preferable over bupivacaine as it did not decrease analgesic duration. Our meta-regression showed that an increased dose of long-acting local anesthetic correlated with increased block duration. Therefore, it is possible to mitigate the reduced duration of blockade by increasing the dose of long-acting local anesthetic in the mixture, but practitioners are advised to be mindful of the total dose of local anesthetics to avoid toxicity. As for using epinephrine, it did not mitigate the decreased block duration, and with the potential for elevated neurotoxicity, we do not recommend its addition in mixed local anesthetics to hasten block onset or prolong duration.

### Study limitations

Despite using strict criteria to achieve a homogenous study population, the statistical heterogeneity is very high in our pooled analysis. This heterogeneity persisted in subgroup analysis for both the primary and secondary outcomes. Therefore, our findings should be interpreted with caution. Several subgroup analyses included only a small number of studies, and most did not show an in-between group difference. There were also only 3 studies investigating mepivacaine and 4 studies investigating the effect of mixing local anesthetics on lower limb blocks.

Varying combinations of local anesthetic mixtures were identified as a potential contributor to heterogeneity; no other significant contributors to heterogeneity were identified in our subgroup or sensitivity analyses. It is likely that variability in block types, techniques, local anesthetic regimen, definition of endpoints, surgical interventions, and patient populations all contributed to heterogeneity. It is not possible to reconcile this without excluding a significant number of studies. Further, there were many small studies with large standard errors, leading to small-study effect, further compounding heterogeneity. Future studies should have larger sample sizes and focus on standardizing these parameters. Despite the heterogeneity, the reduced block latency and duration remained consistent in subgroup analysis. Given the extremely high heterogeneity, we rank the certainty of evidence as low to very low.

## Conclusions

Our systematic review and meta-analysis found that using a mixture of short- and long-acting local anesthetics modestly reduce the time to surgical blockade, with associated decreases in the duration of sensory and motor block, but not analgesic duration. This advantage may be outweighed by shortened block duration. In providers with limited ultrasound experience or where ultrasound is unavailable, this approach may be beneficial where rapid block onset is important, particularly in lower limb blocks. If prolonged analgesia is desired, mixed local anesthetics should not be used. Future study should evaluate the role of mixed LA in lower limb blocks and optimal dosing of long-acting LA to balance onset latency and analgesic duration.

## Funding

This study did not receive any funding.

## Conflicts of interest

The authors declare no conflicts of interest.

## Appendix Supplementary materials

Image, application 1

## Footnotes

Supplementary material associated with this article can be found in the online version at doi:10.1016/j.bjane.2025.844617.
