# Supplementary material for: Comparing block characteristics of mixtures of short/intermediate- and long-acting local anesthetics for peripheral nerve block: a systematic review and meta-analysis
Source: Braz J Anesthesiol. 2025 Mar 28;75(3):844617. doi: 10.1016/j.bjane.2025.844617 (PMC12018993; doi:10.1016/j.bjane.2025.844617)
Supplement: Supplementary file 1 [file mmc1.docx]

**BJAN-D-24-00484_ Supplementary Materials**

**Supplemental Digital Content 1**

| **Search strategy** | |
| --- | --- |
| 1 | exp Drug Combinations/ or exp Drug Therapy, Combination/ or (polytherap$ or combination$ or polychemotherap$ or mixture$ or mixed-to or mix-of or mixed or admixture$ or mixing or ((adding or addition of) adj4 to) or plus or adjuvant$).tw, kf. (2629525) |
| 2 | exp Peripheral Nerves/ or ((median or ulnar or radial) adj2 nerve$).tw, kf. or (endoneurium$ or epineurium$ or peripheral$ or perineurium$).tw, kf. (886141) |
| 3 | (paravertebral$ or femoralsciatic$ or femoral$ or sciatic$ or (brachial$ adj3 plexus$) or supraclavicular$).tw, kf. (208718) |
| 4 | exp Lower Extremity/ or exp Upper Extremity/ or ((extremit$ or limb$ or foot or feet or hip$1 or coxa$1 or knee$1 or legs or leg or ankle$ or thigh$1 or arm or arms or forearm$ or hand$1 or hands$1 or wrist$1 or shoulder$ or neck$1 or orthopedic$ or orthopaedic$ or femur$ or femoral$ or tibia$ or fibula or talus$ or calf$) adj5 (operat$ or surg$ or arthroscop$ or pre-surg$ or presurg$ or pre-op$ or preop$ or inject$ or upper lower)).tw, kf. (529024) |
| 5 | or/2-4 [Concept: Peripheral nerve] (1507468) |
| 6 | exp *Anesthesia, Conduction/or ((anesthe$ or anaesth$ or block$) adj2 conduction$).tw, kf. (60659) |
| 7 | exp Anesthesia, Local/ or ((anesthe$ or anaesth$) adj2 (local or regional$)).tw, kf. (69189) |
| 8 | exp Nerve Block/ or ((nerve$ or local or sensory$) adj2 block$).tw, kf. or (blockade$ or block$1).tw, kf. (516646) |
| 9 | or/6-8 (583226) |
| 10 | 1 and 5 and 9 (6924) |
| 11 | exp "Hypnotics and Sedatives"/or exp Sensory System Agents/or exp Analgesics, Opioid/ (800988) |
| 12 | exp Bupivacaine/ or (bupivacaine$ or buvacaina or carbostesin or dolanaest or marcain$ or sensorcaine$ or levobupivacaine$ or chirocaine$).mp. (19094) |
| 13 | Ropivacaine/ or (naropeine or naropin or ropivacaine$).mp. (6265) |
| 14 | or/12-13 [Long acting local] (23845) |
| 15 | (chloroprocaine$ or 2-chloroprocaine$ or Nesacaine$).mp. (546) |
| 16 | Mepivacaine/ or (carbocaine or isocaine or isogaine or meaverin or mecain or mepihexal or mepivacain or mepivastesin or polocaine or scandicain or scandicaine or scandinibsa or scandonest$).mp. (2122) |
| 17 | exp Lidocaine/ or (Lidocaine or Dalcaine or lignocaine or octocaine or xylesthesin or xylocaine or xylocitin or xyloneural).mp. (36749) |
| 18 | or/15-17 [Short acting local] (38592) |
| 19 | (pharmacologic$ adj2 agent$).tw, kf. (17924) |
| 20 | (rapid-onset$ or short-acting or shortacting or long-acting or longacting).tw, kf. (45798) |
| 21 | or/11-20 (869997) |
| 22 | 1 and 5 and 9 and 21 (3225) |
| 23 | ((anesthe$ or anaesth$ or block$) adj2 (local or regional$)).ti. and 20 (326) |
| 24 | 22 or 23 (3516) |
| 25 | time factors/or (duration or time$).mp. or outcome$1.tw, kf. [should be safe to filter further] (7637525) |
| 26 | 24 and 25 (2079) |
| 27 | (randomized controlled trial or controlled clinical trial).pt. or randomized.ab. or randomised.ab. or placebo.ab. or drug therapy.fs. or randomly.ab. or trial.ab. or groups.ab. [Box 3.c Cochrane Highly Sensitive Search Strategy for identifying randomized trials in MEDLINE: sensitivity-maximizing version (2008 revision); Ovid format] (5777139) |
| 28 | 26 and 27 (1565) |

**Supplemental Digital Content 2**

**Supplemental Table 1** Reported outcomes by study.

|  | **Time to complete block** | **Time to complete sensory block** | **Time to complete motor block** | **Time to any sensory change** | **Time to any motor change** | **Sensory block duration** | **Motor block duration** | **Analgesic duration** | **Analgesic use** | **Pain score** | **Plasma local anesthetic level** | **Local Anesthetic Toxicity** | **Hemodynamic parameters** | **Intraoperative sedation** | **Satisfaction** | **Failure** | **Neurologic deficit** |
| --- | --- | --- | --- | --- | --- | --- | --- | --- | --- | --- | --- | --- | --- | --- | --- | --- | --- |
| Abdelhady [3] |  | ✓ |  | ✓ | ✓ | ✓ | ✓ | ✓ |  | ✓ |  |  |  |  |  |  | ✓ |
| Almasi [10] |  | ✓ | ✓ |  |  | ✓ |  |  |  | ✓ |  |  |  | ✓ |  | ✓ |  |
| Aguilera [9] | ✓ |  |  |  |  | ✓ | ✓ | ✓ |  | ✓ |  | ✓ | ✓ |  |  | ✓ |  |
| Bobik [11] |  | ✓ |  |  |  | ✓ | ✓ |  |  |  |  |  |  |  |  | ✓ | ✓ |
| Bouaziz [12] |  |  |  |  |  |  | ✓ | ✓ |  |  |  |  |  |  |  |  |  |
| Chen [4] |  | ✓ |  |  |  | ✓ |  |  |  |  | ✓ | ✓ | ✓ | ✓ |  | ✓ |  |
| Cuvillon [12] | ✓ |  |  |  |  | ✓ | ✓ | ✓ | ✓ |  |  | ✓ |  |  |  | ✓ |  |
| Freitag [14] |  | ✓ | ✓ |  |  | ✓ | ✓ |  |  |  |  | ✓ |  |  |  | ✓ |  |
| Gadsden [2] |  | ✓ | ✓ |  |  |  | ✓ | ✓ |  | ✓ |  |  | ✓ |  |  |  | ✓ |
| Kim [15] |  | ✓ | ✓ |  |  |  |  | ✓ |  |  |  |  |  |  |  |  |  |
| Laigle [16] | ✓ |  |  |  |  | ✓ |  |  | ✓ |  |  |  |  |  |  | ✓ |  |
| Laur [17] |  | ✓ | ✓ |  |  |  | ✓ |  |  |  |  |  |  |  |  | ✓ |  |
| Martin [18] |  | ✓ |  | ✓ |  | ✓ |  |  |  |  |  | ✓ |  |  |  |  |  |
| Ozmen [19] | ✓ | ✓ |  | ✓ |  |  |  | ✓ |  |  |  | ✓ |  | ✓ |  | ✓ |  |
| Pongraweewan [20] |  | ✓ |  | ✓ | ✓ | ✓ | ✓ |  |  |  |  |  |  |  | ✓ | ✓ |  |
| Rohan [21] |  | ✓ | ✓ | ✓ | ✓ | ✓ | ✓ | ✓ |  |  |  |  |  |  |  |  | ✓ |
| Sripriya [22] | ✓ |  |  |  |  |  |  | ✓ |  |  |  |  |  |  |  | ✓ |  |
| Valery [5] |  | ✓ | ✓ |  |  |  |  | ✓ |  |  |  |  |  |  |  |  |  |
| Zupcic [23] |  | ✓ |  | ✓ |  |  |  | ✓ | ✓ | ✓ |  |  | ✓ | ✓ |  |  |  |

**Supplemental Digital Content 3**

**Supplemental Table 2** Onset of surgical block and its subgroup analysis.

|  | **Study (n)** | **Long-acting local only (n)** | **Mixture (n)** | **MD (min)** | **95% CI** | **p** | **I^2^** | **95% CI** | **P_subgroup_** |
| --- | --- | --- | --- | --- | --- | --- | --- | --- | --- |
| **Time to Complete Block** | | | | | | | | |  |
| Overall | 18 | 505 | 521 | -8.4 | -12.0 to -4.8 | 0.0001 | 0.993 | 0.992 – 0.994 |  |
| **Block Technique** | | | | | | | | | 0.068 |
| Ultrasound^a^ | 12 | 341 | 336 | -6.5 | -11.7 to -1.2 | 0.02 | 0.996 | 0.995 – 0.996 |  |
| Stimulator | 6 | 164 | 185 | -13.7 | -15.7 to -7.7 | <0.001 | 0.940 | 0.900 – 0.964 |  |
| **Block Location** | | | | | | | | | 0.013 |
| Upper limb | 13 | 350 | 384 | -5.4 | -9.2 to -1.5 | 0.010 | 0.969 | 0.958 – 0.977 |  |
| Lower limb | 4 | 115 | 97 | -14.7 | -22.5 to -6.9 | 0.006 | 0.937 | 0.882 – 0.966 |  |
| **Epinephrine used** | | | | | | | | | 0.46 |
| Yes | 8 | 228 | 204 | -7.1 | -10.7 to -3.4 | 0.010 | 0.822 | 0.675 – 0.903 |  |
| No | 10 | 277 | 317 | -9.5 | -16.1 to -2.9 | 0.002 | 0.997 | 0.996 – 0.997 |  |
| **Long-acting local anesthetic in mixture** | | | | | | | | | 0.14 |
| Bupivacaine | 11 | 274 | 310 | -6.3 | -10.5 to -2.1 | 0.007 | 0.957 | 0.940 – 0.970 |  |
| Ropivacaine | 7 | 170 | 171 | -13.3 | -21.1 to -5.5 | 0.005 | 0.977 | 0.967 – 0.984 |  |
| **Short-acting local anesthetic in mixture** | | | | | | | | | 0.027 |
| Lidocaine | 13 | 367 | 388 | -9.3 | -13.1 to –5.5 | <0.0001 | 0.995 | 0.994 – 0.995 |  |
| Mepivacaine | 3 | 67 | 63 | -4.4 | -20.7 to 11.9 | 0.37 | 0.961 | 0.918 – 0.982 |  |
| **Risk of Bias** |  |  |  |  |  |  |  |  | 0.37 |
| Low | 10 | 298 | 315 | -8.4 | -14.2 to -2.7 | 0.008 | 0.982 | 0.976 – 0.986 |  |
| Some concerns | 3 | 90 | 90 | -11.7 | -22.7 to -0.7 | 0.045 | 0.560 | 0.000 – 0.874 |  |
| High | 5 | 116 | 117 | -6.3 | -14.4 to 1.7 | 0.095 | 0.978 | 0.965 – 0.986 |  |
| **Country development level** | | | | | | | | | 0.48 |
| Developed | 8 | 252 | 191 | -7.1 | -12.3 to -1.9 | 0.013 | 0.996 | 0.930 – 0.969 |  |
| Developing | 10 | 269 | 314 | -9.5 | -15.3 to -3.7 | 0.005 | 0.953 | 0.995 – 0.997 |  |

^a^ Include studies using ultrasound and ultrasound with stimulator.

MD, Mean Difference; 95% CI, 95% Confidence Interval; P_subgroup_, p-value for in-between group differences.

**Supplemental Digital Content 4**

**Table 3** Secondary outcomes and subgroup analysis.

|  | **Study (n)** | **Long-acting local only (n)** | **Mixture (n)** | **MD (min)** | **95% CI** | **p** | **I^2^** | **95% CI** | **P_subgroup_** |
| --- | --- | --- | --- | --- | --- | --- | --- | --- | --- |
| **Composite block latency** | | | | | | | | | |
| **Overall** | 6 | 135 | 136 | -11.1 | -14.6 to -7.7 | <0.001 | 0.642 | 0.137 – 0.852 |  |
|  |  |  |  |  |  |  |  |  |  |
| **Ultrasound used** |  |  |  |  |  |  |  |  | 0.44 |
| Yes | 3 | 56 | 56 | -10.0 | -21.5 to 1.5 | 0.064 | 0.774 | 0.266 – 0.930 |  |
| No | 3 | 79 | 80 | -12.2 | -16.5 to –8.0 | 0.006 | 0.0 | 0.000 – 0.896 |  |
|  |  |  |  |  |  |  |  |  |  |
| **Block group** |  |  |  |  |  |  |  |  | 0.97 |
| Upper limb | 3 | 80 | 79 | -11.1 | -24.0 to 1.9 | 0.067 | 0.847 | 0.545 – 0.948 |  |
| Lower limb | 3 | 55 | 57 | -11.1 | -14.1 to –8.2 | 0.003 | 0.0 | 0.000 – 0.896 |  |
|  |  |  |  |  |  |  |  |  |  |
| **Epinephrine used** |  |  |  |  |  |  |  |  | 0.44 |
| Yes | 4 | 81 | 83 | -10.3 | -17.1 to –3.5 | 0.017 | 0.680 | 0.070 – 0.890 |  |
| No | 2 | 54 | 53 | -12.2 | -27.5 to 3.0 | 0.062 | 0.538 | 0.000 – 0.886 |  |
|  |  |  |  |  |  |  |  |  |  |
| **Long-acting local anesthetic used in the mixture** | | | | | | | | | 0.97 |
| Bupivacaine | 5 | 120 | 121 | -11.1 | -16.0 to -6.4 | 0.003 | 0.707 | 0.256 – 0.885 |  |
|  |  |  |  |  |  |  |  |  |  |
| **Short-acting local anesthetic used in the mixture** | | | | | | | | | 0.97 |
| Lidocaine | 5 | 120 | 121 | -11.2 | -16.0 to –6.3 | 0.003 | 0.707 | 0.256 – 0.885 |  |
|  |  |  |  |  |  |  |  |  |  |
| **Risk of bias** |  |  |  |  |  |  |  |  | 0.44 |
| Low | 4 | 81 | 83 | -10.3 | -17.1 to –3.5 | 0.017 | 0.680 | 0.070 – 0.890 |  |
| High | 2 | 54 | 53 | -12.2 | -27.5 to 3.0 | 0.062 | 0.538 | 0.000 – 0.996 |  |
|  |  |  |  |  |  |  |  |  |  |
| **Country development level** |  |  |  |  |  |  |  |  | 0.97 |
| Developed | 3 | 55 | 57 | -11.2 | -14.1 to –8.2 | 0.003 | 0.0 | 0.000 – 0.896 |  |
| Developing | 3 | 80 | 79 | -11.1 | -24.0 to 1.88 | 0.067 | 0.847 | 0.545 – 0.948 |  |
|  |  |  |  |  |  |  |  |  |  |
| **Early sensory block latency** | | | | | | | | |  |
| Overall | 6 | 192 | 189 | -3.7 | -6.7 to -0.6 | 0.027 | 0.985 | 0.978 – 0.989 |  |
| **Ultrasound used** |  |  |  |  |  |  |  |  | 0.0593 |
| Yes | 3 | 108 | 106 | -2.1 | -9.6 to 5.4 |  | 0.994 | 0.990 – 0.994 |  |
| No | 3 | 84 | 83 | -5.6 | -8.6 to -2.6 |  | 0.469 | 0.000 – 0.469 |  |
|  | | | | | | | | | |
| **Block group** |  |  |  |  |  |  |  |  | 0.49 |
| Upper limb | 5 | 152 | 149 | -7.2 | -7.2 to 0.60 | 0.079 | 0.979 | 0.967 – 0.987 |  |
|  | | | | | | | | | |
| **Epinephrine used** |  |  |  |  |  |  |  |  | 0.18 |
| Yes | 2 | 45 | 45 | -5.7 | -23.4 to 11.9 | 0.15 | 0.000 | NA |  |
| No | 4 | 147 | 144 | -3.0 | -7.8 to 1.9 | 0.14 | 0.991 | 0.986 – 0.994 |  |
|  | | | | | | | | | |
| **Long-acting local anesthetic used in the mixture** | | | | | | | | | 0.71 |
| Bupivacaine | 4 | 127 | 124 | -2.9 | -8.5 to 2.8 | 0.20 | 0.978 | 0.963 – 0.987 |  |
|  | | | | | | | | | |
| **Risk of bias** | | | | | | | | | 0.74 |
| Low | 2 | 71 | 69 | -2.4 | -33.9 to 29.0 | 0.51 | 0.988 | 0.976 – 0.994 |  |
| High | 3 | 81 | 80 | -4.1 | -12.8 to 4.7 | 0.18 | 0.980 | 0.964 – 0.989 |  |
|  | | | | | | | | | |
| **Country development level** | | | | | | | | | 0.33 |
| Developing | 5 | 172 | 169 | -3.3 | -6.8 to 0.1 | 0.053 | 0.988 | 0.982 – 0.992 |  |
|  | | | | | | | | | |
| **Early motor block latency** | | | | | | | | |  |
| Overall | 3 | 93 | 91 | -1.3 | -8.4 to 5.7 | 0.50 | 0.976 | 0.953 – 0.987 |  |
|  | | | | | | | | | |
| **Complete sensory block latency** | | | | | | | | |  |
| Overall | 14 | 262 | 299 | -7.8 | -12.6 to -3.0 | 0.004 | 0.995 | 0.994 – 0.996 |  |
|  |  |  |  |  |  |  |  |  |  |
| **Ultrasound used** |  |  |  |  |  |  |  |  | 0.072 |
| Yes | 10 | 300 | 295 | -5.9 | -12.0 to 0.32 | 0.060 | 0.996 | 0.996 – 0.997 |  |
| No | 4 | 104 | 123 | -12.8 | -21.4 to –4.1 | 0.018 | 0.998 | 0.942 – 0.982 |  |
|  |  |  |  |  |  |  |  |  |  |
| **Block group** |  |  |  |  |  |  |  |  | 0.022 |
| Upper limb | 10 | 289 | 323 | -4.4 | -9.0 to 0.3 | 0.061 | 0.974 | 0.965 – 0.982 |  |
| Lower Limb | 3 | 75 | 55 | -16.8 | -35.7 to 2.2 | 0.063 | 0.965 | 0.928 – 0.983 |  |
|  |  |  |  |  |  |  |  |  |  |
| **Epinephrine used** |  |  |  |  |  |  |  |  | 0.063 |
| Yes | 4 | 127 | 101 | -3.5 | -7.9 to 1.0 | 0.088 | 0.849 | 0.625 – 0.939 |  |
| No | 10 | 277 | 317 | -9.5 | -16.1 to –2.9 | 0.010 | 0.997 | 0.996 –0.997 |  |
|  |  |  |  |  |  |  |  |  |  |
| **Long-acting local anesthetic used in the mixture** | | | | | | | | | 0.0011 |
| Bupivacaine | 7 | 193 | 219 | -2.0 | -5.9 to 2.0 | 0.27 | 0.952 | 0.924 – 0.969 |  |
| Ropivacaine | 8 | 171 | 159 | -14.3 | -21.5 to –7.1 | 0.0018 | 0.984 | 0.979 – 0.988 |  |
|  |  |  |  |  |  |  |  |  |  |
| **Short-acting local anesthetic used in the mixture** | | | | | | | | | 0.40 |
| Lidocaine | 9 | 287 | 285 | -8.7 | -15.1 to –2.3 | 0.012 | 0.996 | 0.996 – 0.997 |  |
| Mepivacaine | 3 | 67 | 63 | -3.3 | -20.1 to 13.5 | 0.49 | 0.964 | 0.925 – 0.983 |  |
|  |  |  |  |  |  |  |  |  |  |
| **Risk of bias** |  |  |  |  |  |  |  |  | 0.39 |
| Low | 7 | 217 | 232 | -7.4 | -16.9 to 2.2 | 0.11 | 0.988 | 0.983 – 0.991 |  |
| Some concern | 3 | 90 | 90 | -11.6 | -22.7 to –0.5 | 0.05 | 0.560 | 0.000 – 0.875 |  |
| High | 4 | 97 | 96 | -6.0 | -17.6 to 5.6 | 0.20 | 0.983 | 0.973 – 0.990 |  |
|  |  |  |  |  |  |  |  |  |  |
| **Country development level** | | | | | | | | | 0.40 |
| Developed | 6 | 131 | 190 | -5.6 | -13.8 to 2.6 | 0.14 | 0.961 | 0.937 – 0.976 |  |
| Developing | 8 | 273 | 228 | -9.4 | -16.8 to –2.1 | 0.019 | 0.997 | 0.996 – 0.997 |  |
|  | | | | | | | | | |
| **Complete motor block latency** | | | | | | | | |  |
| Overall | 7 | 181 | 220 | -10.4 | -23.4 to 2.6 | 0.098 | 0.991 | 0.988 – 0.993 |  |
|  |  |  |  |  |  |  |  |  |  |
| **Ultrasound used** |  |  |  |  |  |  |  |  | 0.48 |
| Yes | 5 | 136 | 155 | -7.6 | -24.4 to 9.3 | 0.28 | 0.984 | 0.977 – 0.990 |  |
| No | 2 | 45 | 65 | -17.4 | -174.9 to 140.1 | 0.39 | 0.988 | 0.976 – 0.996 |  |
|  |  |  |  |  |  |  |  |  |  |
| **Block group** |  |  |  |  |  |  |  |  | 0.055 |
| Upper limb | 6 | 141 | 200 | -6.9 | -19.1 to 5.4 | 0.21 | 0.989 | 0.984 – 0.992 |  |
|  |  |  |  |  |  |  |  |  |  |
| **Epinephrine used** |  |  |  |  |  |  |  |  | 0.317 |
| Yes | 3 | 86 | 82 | -5.0 | -7.5 to –2.6 | 0.013 | 0.0 | 0.000 – 0.896 |  |
| No | 4 | 95 | 138 | -14.3 | -43.7 to 15.1 | 0.22 | 0.992 | 0.988 – 0.995 |  |
|  |  |  |  |  |  |  |  |  |  |
| **Long-acting local anesthetic used in the mixture** | | | | | | | | | 0.002 |
| Bupivacaine | 3 | 66 | 105 | 0.23 | -4.4 to 4.9 | 0.89 | 0.888 | 0.740 – 0.987 |  |
| Ropivacaine | 4 | 115 | 115 | -22.1 | -37.2 to –7.0 | 0.013 | 0.990 | 0.986 – 0.992 |  |
|  |  |  |  |  |  |  |  |  |  |
| **Short-acting local anesthetic used in the mixture** | | | | | | | | | 0.59 |
| Lidocaine | 3 | 79 | 102 | -12.8 | -35.3 to 9.8 | 0.36 | 0.996 | 0.995 – 0.997 |  |
| Mepivacaine | 2 | 52 | 48 | -1.0 | -39.1 to 37.1 | 0.80 | 0.830 | 0.293 – 0.959 |  |
|  |  |  |  |  |  |  |  |  |  |
| **Risk of bias** |  |  |  |  |  |  |  |  | 0.62 |
| Low | 5 | 130 | 169 | -13.7 | -32.8 to 5.4 | 0.12 | 0.994 | 0.991 – 0.995 |  |
|  |  |  |  |  |  |  |  |  |  |
| **Country development level** | | | | | | | | | 0.45 |
| Developed | 5 | 116 | 175 | -7.3 | -23.5 to 8.9 | 0.28 | 0.972 | 0.954 – 0.983 |  |
| Developing | 2 | 65 | 45 | -18.0 | -181.9 to 145.9 | 0.40 | 0.994 | 0.989 – 0.997 |  |
|  | | | | | | | | | |
| **Duration of sensory block** | | | | | | | | |  |
| Overall | 12 | 309 | 348 | -226.2 | -352.2 to -100.1 | 0.002 | 0.976 | 0.969 – 0.982 |  |
|  |  |  |  |  |  |  |  |  |  |
| **Ultrasound used** |  |  |  |  |  |  |  |  | 0.34 |
| Yes | 6 | 158 | 177 | -165.6 | -417.7 to 86.5 | 0.15 | 0.956 | 0.926 – 0.973 |  |
| No | 6 | 151 | 161 | -279.8 | -444.0 to –115.5 | 0.006 | 0.984 | 0.977 – 0.989 |  |
|  |  |  |  |  |  |  |  |  |  |
| **Block group** |  |  |  |  |  |  |  |  | 0.34 |
| Upper limb | 10 | 249 | 286 | -202.9 | -363.5 to –42.3 | 0.019 | 0.981 | 0.975 – 0.986 |  |
| Lower limb | 2 | 60 | 62 | -308.1 | -666.2 to 60.0 | 0.066 | 0.833 | 0.493 – 0.945 |  |
|  |  |  |  |  |  |  |  |  |  |
| **Epinephrine used** | | | | | | | | | 0.14 |
| Yes | 6 | 172 | 150 | -304.3 | -498.6 to -110.1 | 0.009 | 0.957 | 0.933 – 0.973 |  |
| No | 6 | 137 | 198 | -142.5 | -338.1 to 53.0 | 0.12 | 0.981 | 0.972 – 0.987 |  |
|  |  |  |  |  |  |  |  |  |  |
| **Long-acting local anesthetic used in the mixture** | | | | | | | | | 0.82 |
| Bupivacaine | 8 | 188 | 218 | -234.8 | -379.6 to -90.1 | 0.005 | 0.934 | 0.898 – 0.957 |  |
| Ropivacaine | 4 | 218 | 130 | -214.4 | -359.3 to -69.5 | 0.010 | 0.981 | 0.974 – 0.987 |  |
|  |  |  |  |  |  |  |  |  |  |
| **Short-acting local anesthetic used in the mixture** | | | | | | | | | 0.006 |
| Lidocaine | 9 | 248 | 269 | -187.8 | -285.6 to -90.2 | 0.011 | 0.958 | 0.942– 0.969 |  |
| Mepivacaine | 2 | 41 | 39 | -204.4 | -2368.5 to 1959.7 | 0.44 | 0.911 | 0.683 – 0.975 |  |
|  |  |  |  |  |  |  |  |  |  |
| **Long-acting local anesthetic dose used in mixture compared to long-acting local comparator** | | | | | | | | | 0.026 |
| Same | 3 | 59 | 85 | - 80.3 | -299.6 to 139.1 | 0.26 | 0.977 | 0.957 – 0.988 |  |
| Lower | 9 | 250 | 246 | -271.7 | -428.0 to -115.5 | 0.003 | 0.970 | 0.957 – 0.978 |  |
|  |  |  |  |  |  |  |  |  |  |
| **Risk of bias** |  |  |  |  |  |  |  |  | 0.14 |
| Low | 8 | 232 | 271 | -265.1 | -454.1 to -76.1 | 0.012 | 0.981 | 0.974 – 0.986 |  |
| Some concern | 2 | 40 | 40 | -188.1 | -480.2 to 103.9 | 0.077 | 0.0 | NA |  |
| High | 2 | 37 | 37 | -107.8 | -663.7 to 448.1 | 0.25 | 0.983 | 0.971 – 0.992 |  |
|  |  |  |  |  |  |  |  |  |  |
| **Country development level** |  |  |  |  |  |  |  |  | 0.32 |
| Developed | 6 | 135 | 198 | -281.7 | -435.6 to -128.0 | 0.004 | 0.977 | 0.966 – 0.984 |  |
| Developing | 6 | 174 | 150 | -162.7 | -423.2 to 97.8 | 0.17 | 0.980 | 0.967 – 0.986 |  |
|  | | | | | | | | | |
| **Duration of motor block** | | | | | | | | |  |
| Overall | 10 | 278 | 273 | -259.2 | -399.5 to -119.0 | 0.002 | 0.982 | 0.977 – 0.986 |  |
|  |  |  |  |  |  |  |  |  |  |
| **Ultrasound used** |  |  |  |  |  |  |  |  | 0.83 |
| Yes | 6 | 176 | 149 | -273.1 | -544.4 to -1.8 | 0.049 | 0.919 | 0.852 – 0.956 |  |
| No | 4 | 102 | 124 | -244.9 | -447.7 to 42.2 | 0.028 | 0.992 | 0.988 – 0.994 |  |
|  |  |  |  |  |  |  |  |  |  |
| **Epinephrine use** | | | | | | | | | 0.38 |
| Yes | 5 | 152 | 129 | -309.9 | -540.1 to -78.9 | 0.018 | 0.967 | 0.947 – 0.979 |  |
| No | 5 | 126 | 144 | -198.9 | -447.32to 49.6 | 0.09 | 0.977 | 0.964 – 0.986 |  |
|  |  |  |  |  |  |  |  |  |  |
| **Block group** |  |  |  |  |  |  |  |  | 0.56 |
| Upper limb | 9 | 238 | 231 | -251.7 | -430.0 to -73.5 | 0.012 | 0.986 | 0.980 – 0.989 |  |
|  |  |  |  |  |  |  |  |  |  |
| **Long-acting local anesthetic used in the mixture** | | | | | | | | | 0.63 |
| Bupivacaine | 8 | 192 | 178 | -288.4 | -449.4 to -127.4 | 0.003 | 0.887 | 0.807 – 0.934 |  |
| Ropivacaine | 2 | 86 | 95 | -240.9 | -423.5 to -58.3 | 0.019 | 0.992 | 0.989 – 0.994 |  |
|  |  |  |  |  |  |  |  |  |  |
| **Short-acting local anesthetic used in the mixture** | | | | | | | | | 0.024 |
| Lidocaine | 7 | 211 | 189 | -208.6 | -330.2 to -86.9 | 0.003 | 0.956 | 0.937 – 0.969 |  |
| Mepivacaine | 2 | 47 | 44 | -442.6 | -2070.0 to 1184.8 | 0.18 | 0.603 | 0.000 – 0.907 |  |
|  |  |  |  |  |  |  |  |  |  |
| **Risk of Bias** |  |  |  |  |  |  |  |  | 0.60 |
| Low | 7 | 218 | 213 | -273.7 | -477.9 to -69.4 | 0.016 | 0.987 | 0.983 – 0.990 |  |
| High | 3 | 60 | 60 | -221.3 | -387.8 to -54.8 | 0.029 | 0.490 | 0.000 – 0.851 |  |
|  |  |  |  |  |  |  |  |  |  |
| **Country development level** |  |  |  |  |  |  |  |  | 0.12 |
| Developed | 5 | 124 | 143 | -349.9 | -472.1 to -227.6 | < 0.001 | 0.776 | 0.503 – 0.899 |  |
| Developing | 5 | 154 | 130 | -157.9 | -471.7 to 155.8 | 0.23 | 0.979 | 0.968 – 0.987 |  |
|  |  |  |  |  |  |  |  |  |  |
|  |  |  |  |  |  |  |  |  |  |
| **Analgesic duration** | | | | | | | | |  |
| Overall | 11 | 295 | 296 | -130.5 | -265.9 to 4.9 | 0.057 | 0.978 | 0.971 – 0.984 |  |
|  |  |  |  |  |  |  |  |  |  |
| **Ultrasound used** |  |  |  |  |  |  |  |  | 0.16 |
| Yes | 8 | 233 | 212 | -165.1 | -355.8 to 25.6 | 0.080 | 0.971 | 0.957 – 0.980 |  |
| No | 3 | 82 | 84 | -38.9 | -212.0 to 134.2 | 0.44 | 0.349 | 0.000 – 0.789 |  |
|  |  |  |  |  |  |  |  |  |  |
| **Block group** |  |  |  |  |  |  |  |  | 0.62 |
| Upper limb | 8 | 195 | 194 | -159.3 | -361.6 to 43.1 | 0.10 | 0.984 | 0.977 – 0.988 |  |
| Lower limb | 2 | 80 | 62 | -56.9 | -818.3 to 704.4 | 0.52 | 0.653 | 0.000 – 0.921 |  |
|  |  |  |  |  |  |  |  |  |  |
| **Endpoint definition** |  |  |  |  |  |  |  |  | 0.99 |
| Onset of pain | 6 | 151 | 131 | -131.1 | -241.1 to -21.3 | 0.028 | 0.948 | 0.912 – 0.970 |  |
| Analgesic request | 4 | 124 | 125 | -150.4 | -718.8 to 418.0 | 0.46 | 0.942 | 0.883 – 0.971 |  |
|  |  |  |  |  |  |  |  |  |  |
| **Epinephrine used** | | | | | | | | | 0.43 |
| Yes | 4 | 117 | 115 | -214.6 | -702.9 to 273.7 | 0.26 | 0.972 | 0.959 – 0.982 |  |
| No | 7 | 200 | 179 | -78.0 | -204.9 to 30.9 | 0.12 | 0.935 | 0.865 – 0.968 |  |
|  |  |  |  |  |  |  |  |  |  |
| **Long-acting local anesthetic used in the mixture** | | | | | | | | | 0.51 |
| Bupivacaine | 7 | 160 | 160 | -250.2 | -479.4 to -21.1 | 0.036 | 0.954 | 0.930 – 0.971 |  |
| Ropivacaine | 3 | 115 | 96 | -119.2 | -277.3 to 38.8 | 0.11 | 0.876 | 0.753 – 0.937 |  |
|  |  |  |  |  |  |  |  |  |  |
| **Short-acting local anesthetic used in the mixture** | | | | | | | | | 0.98 |
| Lidocaine | 9 | 264 | 245 | -184.4 | -382.5 to -40.4 | 0.016 | 0.975 | 0.968 – 0.981 |  |
|  |  |  |  |  |  |  |  |  |  |
| **Risk of Bias** |  |  |  |  |  |  |  |  | 0.65 |
| Low | 5 | 146 | 128 | -187.4 | -543.2 to 168.4 | 0.22 | 0.933 | 0.874 – 0.964 |  |
| Some concern | 2 | 70 | 70 | -132.7 | -494.7 to 229.3 | 0.13 | 0.299 | NA |  |
| High | 4 | 99 | 98 | -67.9 | -308.3 to 172.6 | 0.44 | 0.975 | 0.957 – 0.985 |  |
|  |  |  |  |  |  |  |  |  |  |
| **Country development level** |  |  |  |  |  |  |  |  | 0.93 |
| Developed | 4 | 108 | 110 | -126.6 | -271.6 to 18.5 | 0.069 | 0.013 | 0.000 – 0.940 |  |
| Developing | 7 | 207 | 186 | -136.1 | -368.9 to 96.8 | 0.20 | 0.987 | 0.982– 0.990 |  |
|  |  |  |  |  |  |  |  |  |  |
| **Long-acting local anesthetic dose used in mixture compared to long-acting local comparator** | | | | | | | | | 0.25 |
| Same | 3 | 75 | 75 | -51.1 | -281.3 to 179.1 | 0.44 | 0.827 | 0.470 – 0.943 |  |
| Lower | 8 | 220 | 221 | -165.1 | -358.5 to 28.4 | 0.08 | 0.962 | 0.943 – 0.975 |  |

MD, IMean Difference; 95% CI, 95% Confidence interval; P_subgroup_, p-value for in-between group differences.
